# Polyurethane scaffold-based 3D lung cancer model recapitulates *in vivo* tumor biological behavior for nanoparticulate drug screening

**DOI:** 10.1093/rb/rbad091

**Published:** 2023-10-25

**Authors:** Lu Sun, Xiaofei Wang, Yushui He, Boran Chen, Baoyin Shan, Jinlong Yang, Ruoran Wang, Xihang Zeng, Jiehua Li, Hong Tan, Ruichao Liang

**Affiliations:** Department of Targeting Therapy & Immunology; Department of Radiation Oncology, Cancer Center, West China Hospital, Sichuan University, Chengdu 610041, People's Republic of China; Department of Medical Polymer Materials; Department of Artificial Organism, College of Polymer Science and Engineering, State Key Laboratory of Polymer Materials Engineering, Sichuan University, Chengdu 610065, People’s Republic of China; Department of Medical Polymer Materials; Department of Artificial Organism, College of Polymer Science and Engineering, State Key Laboratory of Polymer Materials Engineering, Sichuan University, Chengdu 610065, People’s Republic of China; Department of Neurosurgery, West China Hospital, Sichuan University, Chengdu 610041, People’s Republic of China; Department of Neurosurgery, West China Hospital, Sichuan University, Chengdu 610041, People’s Republic of China; Department of Neurosurgery, West China Hospital, Sichuan University, Chengdu 610041, People’s Republic of China; Department of Neurosurgery, West China Hospital, Sichuan University, Chengdu 610041, People’s Republic of China; Department of Neurosurgery, West China Hospital, Sichuan University, Chengdu 610041, People’s Republic of China; Department of Medical Polymer Materials; Department of Artificial Organism, College of Polymer Science and Engineering, State Key Laboratory of Polymer Materials Engineering, Sichuan University, Chengdu 610065, People’s Republic of China; Department of Medical Polymer Materials; Department of Artificial Organism, College of Polymer Science and Engineering, State Key Laboratory of Polymer Materials Engineering, Sichuan University, Chengdu 610065, People’s Republic of China; Department of Neurosurgery, West China Hospital, Sichuan University, Chengdu 610041, People’s Republic of China

**Keywords:** WBPU, scaffolds, 3D culture, lung cancer, biomimetic, nanoparticulate drug screening

## Abstract

Lung cancer is the leading cause of cancer mortality worldwide. Preclinical studies in lung cancer hold the promise of screening for effective antitumor agents, but mechanistic studies and drug discovery based on 2D cell models have a high failure rate in getting to the clinic. Thus, there is an urgent need to explore more reliable and effective *in vitro* lung cancer models. Here, we prepared a series of three-dimensional (3D) waterborne biodegradable polyurethane (WBPU) scaffolds as substrates to establish biomimetic tumor models *in vitro*. These 3D WBPU scaffolds were porous and could absorb large amounts of free water, facilitating the exchange of substances (nutrients and metabolic waste) and cell growth. The scaffolds at wet state could simulate the mechanics (elastic modulus ∼1.9 kPa) and morphology (porous structures) of lung tissue and exhibit good biocompatibility. A549 lung cancer cells showed adherent growth pattern and rapidly formed 3D spheroids on WBPU scaffolds. Our results showed that the scaffold-based 3D lung cancer model promoted the expression of anti-apoptotic and epithelial–mesenchymal transition-related genes, giving it a more moderate growth and adhesion pattern compared to 2D cells. In addition, WBPU scaffold-established 3D lung cancer model revealed a closer expression of proteins to *in vivo* tumor, including tumor stem cell markers, cell proliferation, apoptosis, invasion and tumor resistance proteins. Based on these features, we further demonstrated that the 3D lung cancer model established by the WBPU scaffold was very similar to the *in vivo* tumor in terms of both resistance and tolerance to nanoparticulate drugs. Taken together, WBPU scaffold-based lung cancer model could better mimic the growth, microenvironment and drug response of tumor *in vivo*. This emerging 3D culture system holds promise to shorten the formulation cycle of individualized treatments and reduce the use of animals while providing valid research data for clinical trials.

## Graphical abstract

**Figure:**
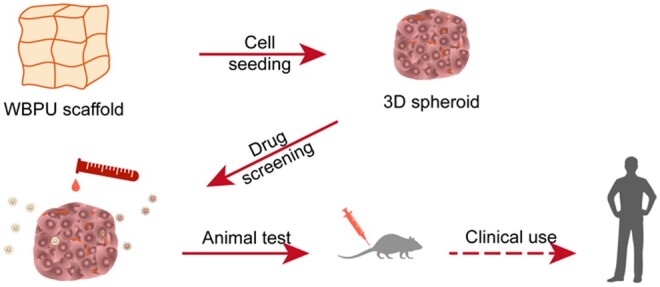

## Introduction

Lung cancer is one of the world’s most challenging oncologic diseases, which accounts for nearly 20% of cancer deaths worldwide [1]. In addition to surgical resection and radiation therapy, chemotherapy and targeted therapy are currently the mainstays of lung cancer treatment. Although new drugs are constantly being developed, low drug bioavailability and the lack of effective drug evaluation models are major problems in the clinical translation of drugs [2]. Nanomedicine is considered to be a revolutionary change in lung cancer therapy, which greatly improves drug solubility, prolongs drug circulation and distribution in the body, and greatly reduces the toxic effects of chemotherapeutic agents [3–5]. However, the abnormally elevated interstitial pressure in solid tumors and the pathological barrier formed by the highly dense extracellular matrix (ECM) make it difficult for nanoparticles (NPs) to penetrate deeply into the tumor, resulting in compromised drug efficacy [6]. These limitations have led to significant challenges in the clinical application of nanocarriers in lung cancer. To date, only a few antitumor-targeting nanocarriers have been approved by the Food and Drug Administration for clinical use [3]. Therefore, the selection of an effective *in vitro* evaluation system to verify the potency of the developed drug carriers is an important issue.

Currently, most antitumor drug or drug vector evaluation systems are still the traditional *in vitro* two-dimensional (2D) cell models and *in vivo* xenograft tumor models [2]. Lung cancer cells cultured in 2D cannot truly reflect the *in vivo* tumor status, leading to large discrepancies between *in vitro* experiments and *in vivo* evaluations [7]. Due to high reproducibility and preservation of some biological features of human tumors, immunodeficient mouse subcutaneous xenograft tumor models are widely used in oncology research for drug testing [8]. However, the production of animal models takes a lot of time. In addition, drug development is time-consuming and dropout rates are high. In preclinical and phase I clinical studies alone, the rate of discontinuation is more than 85% [2]. Indeed, deposition of ECM in 3D tumor models *in vivo* has been shown to impair drug penetration and diffusion, thereby reducing drug efficacy [9]. Reduced drug permeability is one of the most common causes of drug resistance in tumors *in vivo*, and the drug resistance characteristics of 3D cell models are similar to those of solid tumors *in vivo* [10]. Therefore, 3D cell models may be more suitable for drug evaluation and screening.

In recent years, tissue-engineered tumors based on 3D cultures have been widely used in oncology research [11, 12]. 3D models of tumor cells grow in clusters with a gradient distribution of nutrients and oxygen within the tumor sphere. Cells in the central region show quiescent growth and necrosis due to a lack of oxygen and nutrients, while cells in the periphery proliferate actively. This has similar pathological characteristics to solid tumor cells *in vivo* [12]. Therefore, the *in vitro* 3D tumor models can simulate the growth of solid tumors *in vivo*. Recently, 3D models constructed from predefined scaffolds have attracted attention. These scaffold-based 3D models exhibit heterogeneous cell growth conditions and gradient distribution of nutrients and oxygen. Due to these features, they have been widely used to evaluate drug resistance and drug penetration in tumor tissues [11, 12]. The presence of good biocompatibility and favorable cell adhesion and proliferation are key factors for scaffolds to construct 3D models [11]. Scaffold materials currently used for 3D tumor cell culture mainly include synthetic polymer materials such as polylactic acid, polyacrylamide and poly(lactide-co-glycolic acid) [13–15], and natural biomaterials such as matrigel, collagen hydrogel and hyaluronic acid hydrogel [16–18]. Although these 3D scaffold materials have been used for the construction of preclinical tumor models, lung cancer models still need to be further investigated.

Polyurethane (PU) is an emerging organic polymer material. Due to its diverse molecular structure, excellent mechanical properties, biocompatibility and adaptability, it has been widely explored and used as scaffolds, implants, hydrogels and drug carriers in biomedicine [19]. A variety of PU scaffold materials have been fabricated using nanotechnology, electrostatic spinning and reverse spraying for the construction of blood vessels, cartilage and bone in tissue engineering [20–22]. Gupta *et al.* have used PU scaffolds for *in vitro* pancreatic cancer cell culture for drug screening and assessment of tumor response to radiotherapy [23–25]. Therefore, the use of PU scaffolds as the basis for *in vitro* 3D tumor models is promising. In our previous work, we prepared a series of porous 3D waterborne biodegradable polyurethane (WBPU) scaffolds [26, 27]. These scaffolds exhibited good biocompatibility and biodegradability and were shown to promote the repair of neurological defects in brain-injured rats [27]. In addition, we have successfully developed a series of PU NPs and drug-loaded PU scaffolds that can target tumors at multiple levels, increase drug uptake, and effectively inhibit tumor growth [28–33].

In this article, we constructed 3D biomimetic lung cancer models *in vitro* on the basis of 3D porous WBPU scaffolds. The biological properties of the tumor models were evaluated, the tumor permeability of different drug carriers was investigated and the antitumor activity of the drugs was verified. It provides a new strategy for constructing a potential and practical tool for antitumor drug evaluation.

## Materials and methods

### Preparation and characterization of 3D WBPU scaffolds

A series of WBPU was synthesized using polyethylene glycol (PEG, Mw 1450, Dow Chemical, USA), poly-3-caprolactone (PCL, Mn 2000, Dow Chemical, USA), L-lysine (Emei Biochemical, China), 1,3-propanediol (PDO, Kelong, China) and L-lysine diisocyanate (LDI, Dahong Chemical, China). The WBPU emulsions and scaffolds were prepared in accordance with our recent study [26, 31]. Briefly, PEG and PCL were prepolymerized with LDI under a dry nitrogen atmosphere and 1‰ of organic bismuth catalyst at 80°C for 1 h. Then the chain extender PDO was added to react at 65°C for 2 h. Finally, the pre-polyurethane was emulsified by mixing with L-lysine solution for 3 h to obtain polyurethane emulsions. The emulsions were dropped into 96-well plates to fabricate WBPU scaffolds by freeze-drying method in a freeze-dryer (Boyikang, Beijing). The internal pores and structure of the WBPU scaffolds were examined by scanning electron microscopy (SEM) (Quanta 200, Philips, Netherlands), before observation, the scaffolds were fractured in liquid nitrogen and coated with gold. Compressive modulus tests were performed on these WBPU scaffolds using a tensile testing machine (HZ-1004, Lixian Instruments, China). Water absorption rate was characterized using gravimetric analysis and calculated using the following equation:


$$\mathrm{Water}\;\mathrm{absorption}\;(\%)\;=\;\frac{W_{w}-W_{d}}{W_{d}}\;\times \;100\%$$

where *W_w_* and *W_d_* are the wet and dry weights of the WBPU scaffolds, respectively (*n* = 3).

### Cell culture

L929 (mouse fibroblast cell line) and A549 (human non-small-cell lung cancer cell line) were the main experimental cells in this study. All cells were cultured in Dulbecco’s Modified Eagle Medium (DMEM, Gibco) complete medium supplemented with 10% fetal bovine serum (FBS, Gibco) and 1% penicillin-streptomycin double antibiotic (Gibco), respectively. Cell culture conditions were 37°C, 5% CO_2_ atmosphere.

### Cytocompatibility examination

The cytocompatibility of the WBPU scaffold was assessed by culturing L929 cells with the scaffold-soaked medium, and cell viability was examined by the MTT (3-[4,5-dimethylthiazol-2-yl]-2,5 diphenyl tetrazolium bromide) method. Stably propagated and passaged cells were seeded in 96-well plates with 3 × 10^3^ cells per well and cultured until stable cell attachment. The medium was removed and replaced with 100 μl of scaffold-soaked medium, and a complete medium without scaffold-soaking was used as a control. The scaffold-soaked medium was removed after 24, 48 and 72 h of incubation, and L929 cells continued to grow in a medium containing MTT (5 mg/ml) for 4 h. The MTT medium was removed and the formazan crystals in the cells were dissolved with dimethyl sulfoxide. Finally, the absorbance at 570 nm was measured using a microplate reader (DNM-9602, China) and the relative cellular activity was calculated. Meanwhile, the morphology of L929 cells at each time point was collected by an inverted microscope.

### A549 cell growth in the scaffolds

Stably propagated and passaged A549 cells were seeded in WBPU scaffolds (5 × 10^3^ cells per scaffold). After 1, 3, 7 and 14 days of cultivation in DMEM complete medium, scaffolds containing cells were fixed with 4% paraformaldehyde. After permeabilization with 0.2% Triton-X (Sigma, USA) and blocking with 1% bovine serum albumin (BSA, Gibco) and 5% FBS, cells in the scaffolds were stained with rhodamine-labeled phalloidin and DAPI (4′,6-diamidino-2-phenylindole). Finally, the morphology of the cells in the scaffolds was recorded by A1R MP + laser confocal microscopy (Nikon, Japan).

### Lung cancer models growth on the scaffolds

Stably propagated and passaged A549 cells were seeded in PU4 scaffolds (scaffold at 1 mm^3^ size, 1 × 10^4^ cells per scaffold), and cells with the same initial number seeded in blank plates were set as 2D control. Cells in the 2D and 3D systems were cultured in a complete medium for 24 and 48 h, respectively. Phalloidin staining experiments were performed as described in ‘A549 cell growth in the scaffolds’ section. The cell viability of 2D and 3D cultured cells was examined by MTT method described in ‘Cytocompatibility examination’ section.

### Cell cycle and apoptosis analysis

Cell cycle and apoptosis of 2D and 3D cultured A549 cells were examined by flow cytometry. After cultivated in plates or scaffolds for 24 and 48 h, the cells were harvested, washed and finally resuspended in DPBS with RNAse. Cell cycle was tested using propidium iodide (PI) staining, apoptosis was measured using annexin V-FITC and PI double staining as described previously [34]. The analysis was carried out by an ACEA NovoCyte flow cytometer (ACEA Biosciences Inc.), and a negative control was prepared to set the sampling parameters of the flow cytometer and the range of the gate. Data process was performed using FlowJo software.

### Quantitative polymerase chain reaction analysis

The quantitative polymerase chain reaction (qPCR) was performed using a real-time fluorescence quantitative PCR system (CFX opus 96, Biorad, USA). TRIzol (Invitrogen, USA) was used to isolate and extract total mRNA from 2D and 3D cultured A549 cells on 72 h, and after determining the concentration, qPCR reaction was processed as follows: denaturation at 95°C for 30 s, amplification at 95°C for 5 s, 60°C for 30 s, 40 cycles, dissolution at 95°C for 5 s, 60°C for 1 min. The relative expression of genes between 2D and 3D models was analyzed by the 2^−ΔΔCt^ method. GAPDH was chosen as a housekeeping gene, the primer sequences required for the reaction are listed in Supplementary Table S1.

### Subcutaneous and in situ lung cancer models in nude mice

Animal experiments were conducted with authorization from the Sichuan University Ethics Committee (number: 20230824005) and were according to the principles of the National Institute of Health of China for the care of animals in experiments. 4- to 6-week-old female BALB/c nude mice of SPF class (18–20 g) were obtained from China Vital River Laboratory Animal Technology. Mice were housed in an environment with a temperature of 20–22°C, relative humidity of 50–60% and a 12-h light-dark cycle and were provided with sterile food and drinking water. Stably proliferating A549 cells were collected, washed and resuspended in DPBS. Mice received 0.2 ml cells (5 × 10^6^/ml) by subcutaneous injection to construct subcutaneously xenograft tumor model and 0.15 ml cells (1 × 10^5^/ml) by tail vein injection to construct *in situ* lung cancer model.

### Western blot (WB)

Biomolecular markers of different lung cancer models were detected by WB. A549 2D cells and 3D spheroids were collected and resuspended in RIPA lysis buffer (Hangzhou HuaAn Biotechnology Co., Ltd., China) containing PMSF (Hangzhou HuaAn Biotechnology Co., Ltd., China). Subcutaneously xenograft tumor was frozen and homogenized in cold lysis buffer containing PMSF. All samples were continuously agitated for 30 min at 4°C. Then, the lysates were centrifuged at 12 000 rpm for 20 min at 4°C. The supernatants were aspirated and placed in fresh tubes on ice. The protein concentration was determined with the bicinchoninic acid (BCA) assay using the BCA Protein Assay Kit (Thermo Fisher Scientific, USA). Protein lysates were boiled in Laemmli buffer (5×), separated by SDS-PAGE and transferred to PVDF membranes. The membranes were probed with antibodies against GAPDH, OCT4, ALDH1, Musashi-1, BCL-2A1, FN, ANXA1, ErbB3, EGFR, P-GP, LRP, MRP1 and SOX2 (Abcam). After that, the membranes were incubated with horseradish peroxidase-conjugated secondary IgG antibody, and chemiluminescence detection was carried out using the ChemiDoc MP Imaging System (Biorad, USA).

### Uptake and distribution of nanocarriers in lung cancer models

G8mE1900 micelles (size 61.77 ± 1.24 nm, zeta potential 29.83 ± 1.03 mV) and GFHPM micelles (size 78.2 ± 0.7 nm, zeta potential −16.13 ± 1.06 mV) were prepared according to our previous reports [28, 29]. Briefly, as for G8mE1900, polyurethanes bearing gemini quaternary ammoniums (GQAs) with eight carbon number alkyl chains (G8) were synthesized from mPEG (Mn = 1900), PCL, LDI and PDO to obtain G8mE1900 polymers. As for GFHPM, polyurethane incorporated with hydrazone, folic acid (FA), or GQA ligands was mixed with a ratio GQA-PU (2), FA-PU (2), and H-PU (6) to obtain GFHPM (G2F2H6) polyurethane. The G8mE1900 and GFHPM micelles were prepared using the dialysis method. The dimensions and zeta potential of micelles were determined by dynamic light scattering measurements on a Malvern Zetasizer Nano (Zen 3690, Malvern, UK). Both charged nanocarriers have been demonstrated with good biocompatibility. FITC, DOX and PTX were loaded into micelles using the thin-film hydration and dialysis method. Drug-loaded micelles showed steadily controllable drug release. The cellular uptake and distribution of the G8mE1900-FITC and GFHPM-DOX micelles analyzed by fluorescence microscope (Ti2, Nikon).

### Antitumor activity in 2D and 3D lung cancer models

The *in vitro* cytotoxicity of G8mE1900-PTX and GFHPM-DOX micelles was evaluated on A549 cells cultured in 2D and 3D systems by MTT method. Stably propagated and passaged cells were seeded in scaffold cubes (1 × 10^4^ cells per cube) and cultured in a complete medium for 48 h to form 3D spheroids. 2D cells cultured in blank plates were set as control. At the expected time point, the initial medium was removed and replaced with a fresh medium containing various concentrations of G8mE1900-PTX and GFHPM-DOX micelles. At 24, 48, 72 and 120 h after incubation, cell viability was tested by MTT as described in ‘Cytocompatibility examination’ section. Meanwhile, the phalloidin assay was performed as described in ‘A549 cell growth in the scaffolds’ section.

### Antitumor activity in vivo

Antitumor efficacy of G8mE1900-PTX and GFHPM-DOX micelles *in vivo* was investigated using a subcutaneous xenograft tumor model and *in situ* lung cancer model. G8mE1900-PTX micelles (5 mg/kg equivalent PTX) and GFHPM-DOX micelles (4 mg/kg equivalent DOX) were injected by the tail vein every 3 days for a total of 21 days. Subcutaneous tumor volume was estimated according to the equation: Volume = π/6 × length × width^2^. At the endpoint of the experiment, all mice were sacrificed and neoplastic masses were harvested for weight calculation (subcutaneous), lung nodule count (*in situ* lung cancer model) and H&E assay (both).

### Statistical analysis

All data are expressed as mean ± standard deviation. Statistical analysis was performed through paired *t*-tests or one-way analysis of variance with Prism software (8.4.0). Values of *P* < 0.05 were recognized as statistically significant (∗*P *<* *0.05, ∗∗∗*P *<* *0.01, ∗∗∗∗*P *<* *0.001, ∗∗∗∗*P *<* *0.0001, and ns: *P *>* *0.05).

## Results and discussion

### Characterization and biocompatibility of 3D WBPU scaffolds

**Figure 1.**
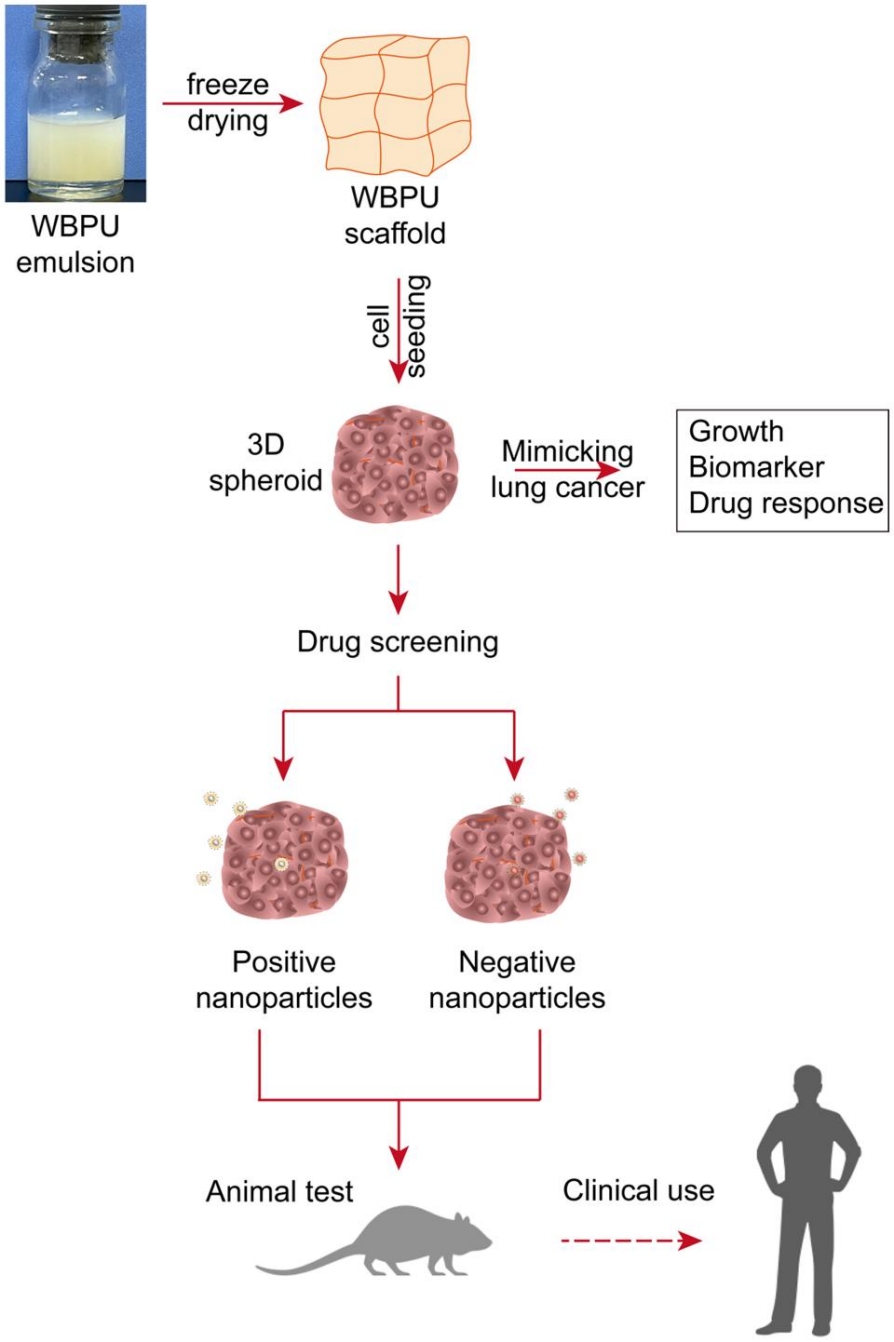
Schematic diagram for preparation of scaffolds, construction and characterization of 3D lung cancer models, 3D model-based drug screening, and subsequent animal testing and clinical applications.

A series of 3D WBPU scaffolds (PU1∼4) with 1-mm thickness and 6-mm diameter were fabricated according to our previous study (Figure 1 and Supplementary Figure S1). Briefly, a multi-segmented PU emulsion consisting of soft segment (PEG, PCL) and hard segment (LDI, PDO) was synthesized by prepolymerization, chain extension and emulsification. Then, the emulsions were lyophilized in the 96-well plate to fabricate scaffolds. All WBPU scaffolds were subjected to gamma sterilization prior to cell and animal studies.

**Figure 2.**
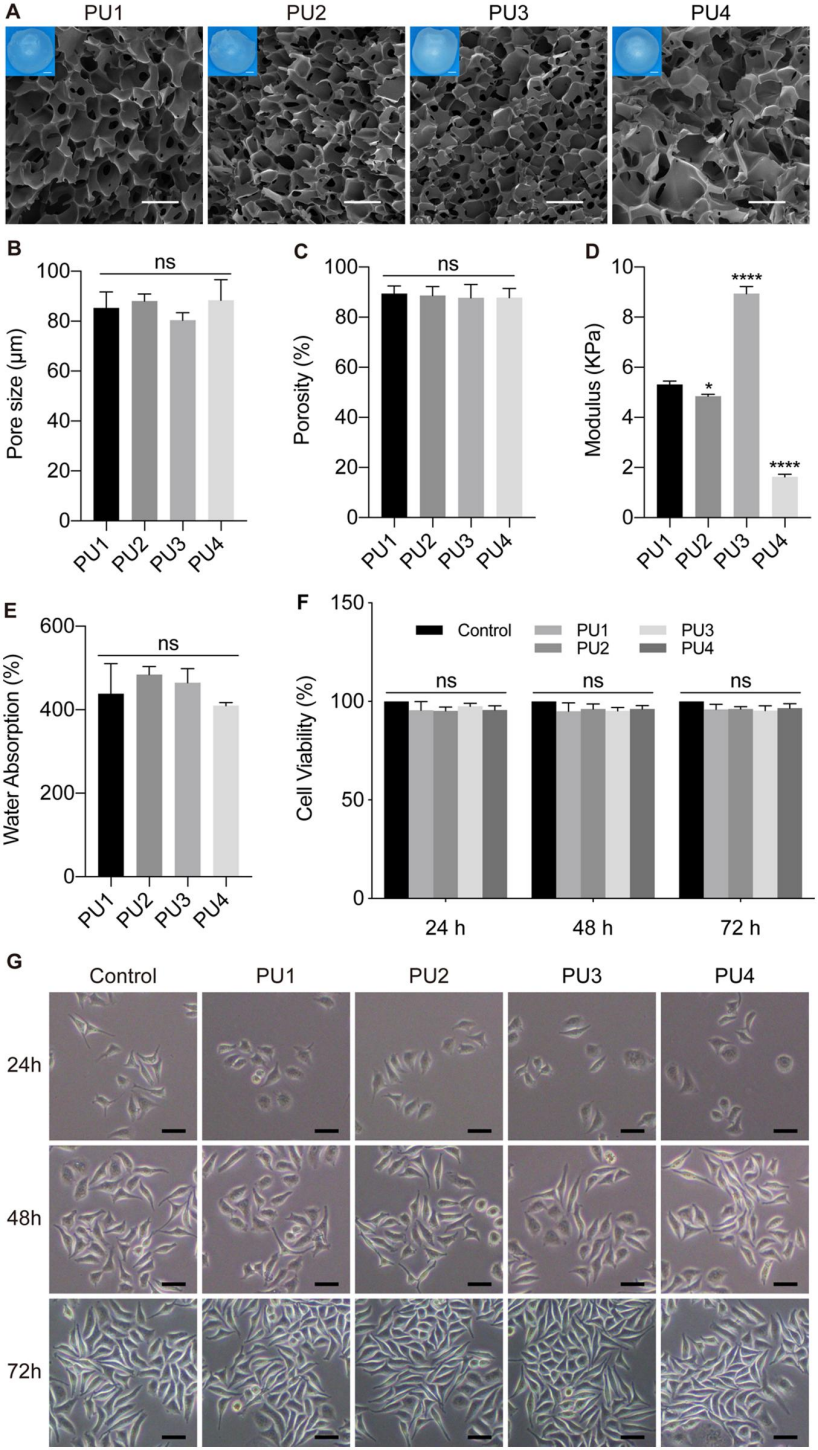
(**A**) Physical photos and SEM images of the lyophilized WBPU scaffolds showing the rich porous structure of the scaffolds, scale bars 1 mm for photos and 100 μm for SEM images. (**B**) The internal pore size of each WBPU scaffold measured by image J was in the range of 80–90 μm, with no statistical difference among scaffolds. (**C**) The internal porosity of each WBPU scaffold measured by image J was in the range of 87–90%, sufficiently ensuring the exchange of material from the cultures on the scaffold. There was no statistical difference in the porosity among the scaffolds. (**D**) The compression modulus of the wet WBPU scaffolds were all below 10 kPa, and the mean modulus of the PU4 scaffold (1.63 kPa) was closest to the mean modulus of the lung tissue (1.9 kPa). (**E**) The water absorption characteristics of the WBPU scaffolds suggested that the scaffolds could store large amounts of free water inside. (**F**) Activity of L929 cells cultured with total scaffold extracts at different time points showed no toxic effects from the scaffolds. (**G**) Morphology of L929 cells incubated with scaffold extracts, scale bar 50 μm. Graphs show mean ± SD. ∗∗∗∗*P* < 0.0001; ns: *P* > 0.05.

The cross-sectional morphology of the lyophilized PU scaffolds was examined using SEM (Figure 2A). All scaffolds exhibited a multiporous structure with high porosity (∼90%, Figure 2C) and uniform pore size (8 0–90 μm, Figure 2B), resembling the alveolar cavity of lung tissue. The modulus of all scaffolds ranged from 1 to 10 kPa (Figure 2D), which was similar to that of lung tissue [35]. The mean modulus of the PU4 scaffold was 1.63 kPa, which was close to the reported modulus of lung tissue of 1.9 kPa. The pores of these scaffolds stored a large amount of free water for cell life activities and information exchange (Figure 2E). To assess the viability of normal cells on the WBPU scaffolds, L929 cells were cultured *in vitro* with total scaffold extracts and cell activity was detected by MTT assay (Figure 2F and G). In general, almost all L929 cells cultured with scaffold extracts for 24, 48 and 72 h showed steady growth (Figure 2G) and relative cell activity above 90% (Figure 2F). These results demonstrate that WBPU scaffolds are suitable for cell growth.

Modification of natural polymeric materials and synthetic polymers has been shown to mimic the ECM [11, 12, 17]. 3D tumor cultures based on such materials may mimic the environment of tumor growth, so that the cultured tumor models more closely resemble the state *in vivo*. To achieve this, the physicochemical properties of the polymeric materials, such as material modulus, should match the texture of *in vivo* tumors. This is because the stiffness of the ECM has been shown to influence tumor cell proliferation, invasion and metastasis in a variety of tumors [36]. Park *et al*. reported that stromal stiffness affects the glycolysis and development of lung cancer [37]. Therefore, the 3D lung cancer model substrates designed in this study first simulated the elastic modulus of lung tissue.

### 3D spheroid culture of human alveolar adenocarcinoma cells in WBPU scaffolds

**Figure 3.**
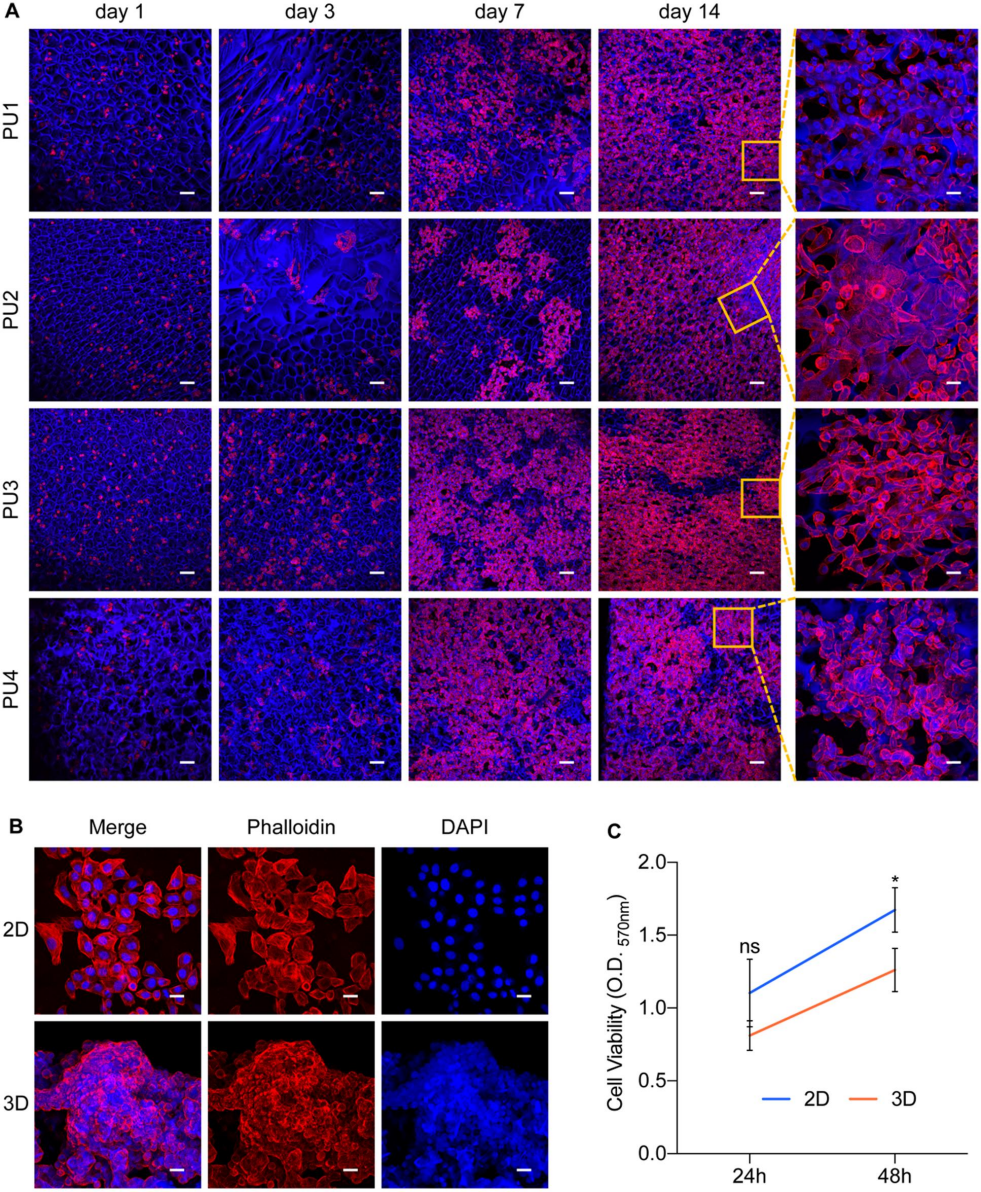
(**A**) 3D Culture of A549 cells in WBPU scaffolds suggested a 3D pattern of colony-like growth of tumor cells on the scaffolds, with spheroid growth of tumor cells on PU4 scaffolds, scale bars 100 μm for low-magnification field and 20 μm for high-magnification field. (**B**) The establishment of 3D spheroids on PU4 scaffolds after 48 h of cultivation, scale bar 20 μm. (**C**) Cell proliferation in 2D- and 3D-cultured A549 cells measured by MTT (*n* = 3). ∗*P* < 0.05; ns: *P* > 0.05.

The human alveolar adenocarcinoma cell line A549 was initially seeded in 3D growth systems on WBPU scaffolds (6-mm disk). It was observed that A549 cells cultured in the scaffolds rapidly formed clonal clusters within 1 day (Figure 3A). After 7 days, the growing clusters became increasingly dense and merged into multilayered structures. After 14 days, cell clusters in PU2, PU3 and PU4 scaffolds covered the entire inner wall of the scaffolds and filled the pores, compared with those in PU1 scaffolds. Notably, lung cancer cells growing in the PU4 scaffolds showed marked intercellular adhesion and formed spheroids (Figure 3A). Previous studies have shown that conventional cell culture materials have high modulus [38]. In the present study, lung cancer cells in PU1, PU2 and PU3 scaffolds with higher modulus had different degrees of elongation, similar to cells cultured in plates. In contrast, almost all cells in the PU4 scaffolds were round spheres that grew around the walls of the scaffold pores, similar to the clinical morphology of lung cancer. Therefore, we hypothesize that the morphology and modulus of the polymer scaffold, which more closely resembles the tissue characteristics of the original tumor site, may better support the establishment of the *in vitro* lung cancer model.

Subsequently, PU4 scaffolds were chosen as the 3D substrates for lung cancer model construction. To investigate the growth rate of the lung cancer model, PU4 scaffolds were cut into 1 mm^3^ cubes for 3D culture of A549 cells. A549 cells with the same initial number (1 × 10^4^/well or cube) were seeded in 96-well plates or scaffold cubes and cultured for 24 and 48 h. Compared with adherent growth in conventional 2D cultures, A549 cells cultured in PU4 scaffolds rapidly formed multilayer spheroids after 48 h (Figure 3B). Cell proliferation was measured by the MTT assay. It was found that the proliferation of 3D spheroids grown on PU4 scaffolds was lower than that of the 2D control (Figure 3C).

### 3D spheroids exhibit unique phenotypes different from 2D system

**Figure 4.**
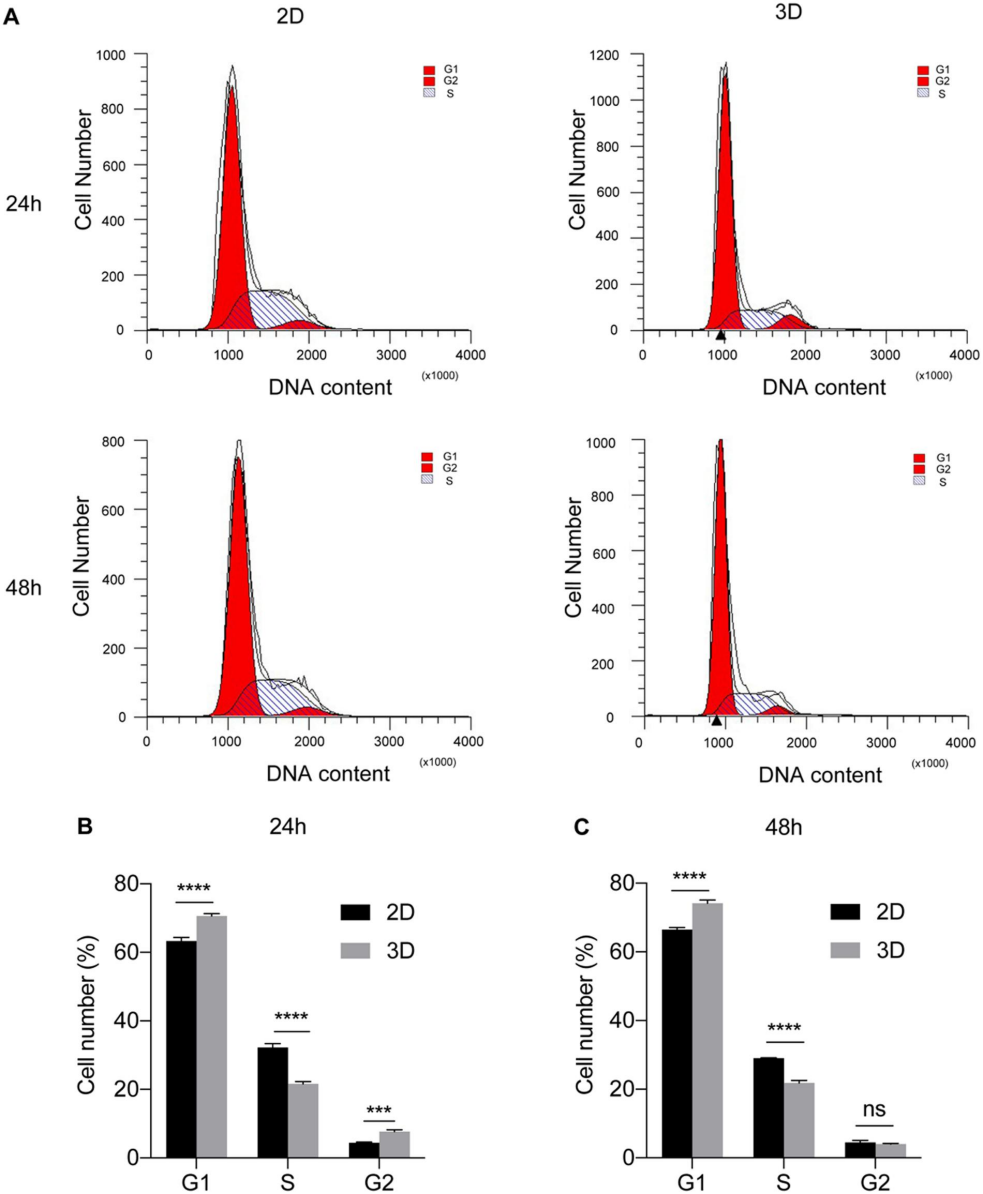
Cell cycle assay of 2D- and 3D-cultured A549 cells. (**A**) Flow cytometry for cell cycle detection at 24 and 48 h after cultivation in each system. Cell number distribution in each cell phase at 24 h (**B**) and 48 h (**C**) in 2D and 3D systems (*n* = 3). The results showed lower division rate of spheroids on scaffolds than cells grown in 2D culture plates. ∗∗∗*P* < 0.001; ∗∗∗∗*P* < 0.0001; ns: *P* > 0.05.

To further explore the proliferation profile of lung cancer cells growing in 2D and 3D systems, we examined the cell cycle of A549 cells by flow cytometry. As shown in Figure 4, A549 spheroids grown for 24 and 48 h in scaffolds showed an increase in G1 (before DNA replication) and G2 (before mitosis) phase and a decrease in S phase (*P* < 0.001) compared with the 2D control. The results indicate that A549 cells have a lower division rate after growth into spheroids on scaffolds than cells grown in 2D culture plates. It is well known that cell culture plates are mainly made of rigid polymers, which provide a uniform and compatible surface for cell adhesion and proliferation [39]. The results of the 3D tumor spheroids we constructed further support this theory.

**Figure 5.**
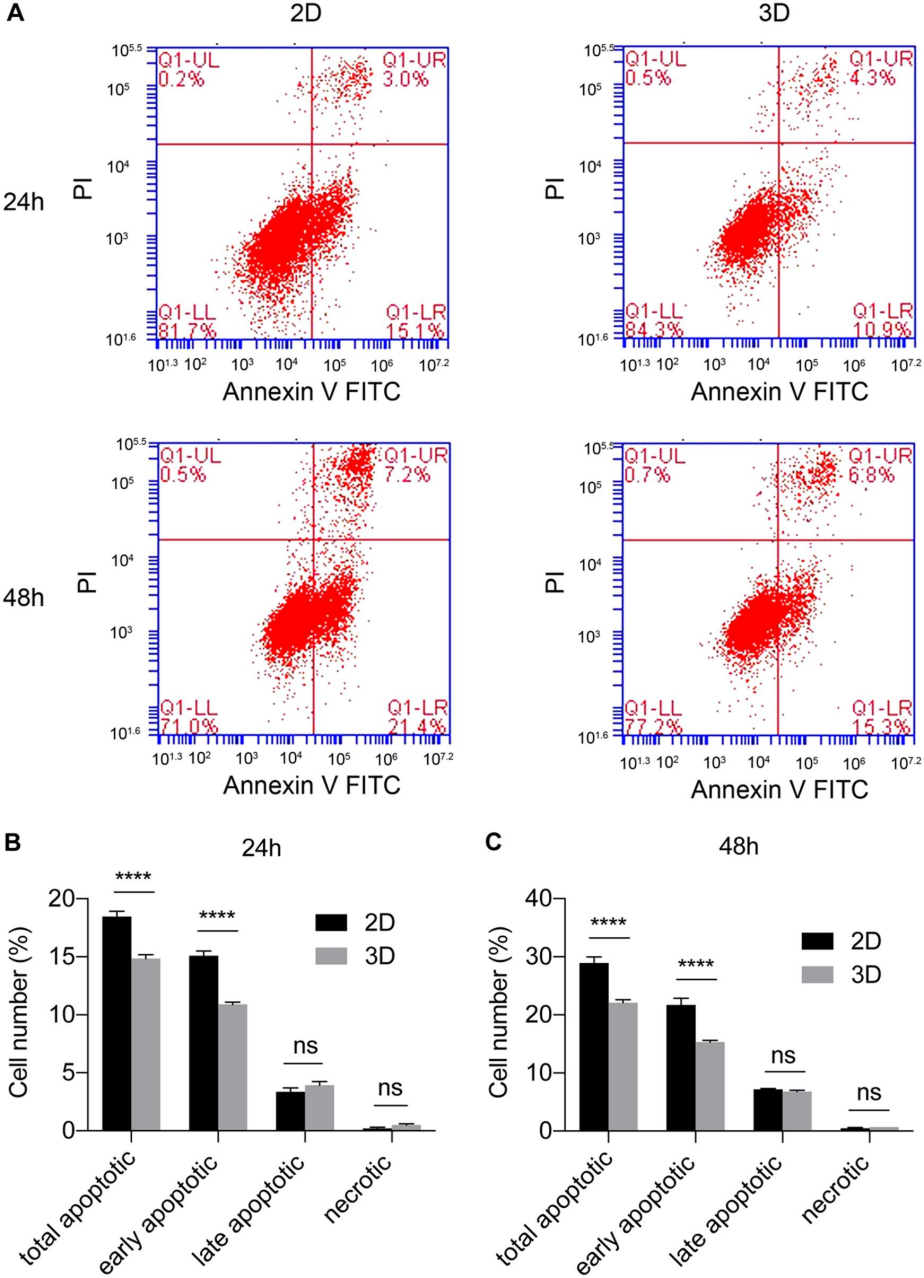
Apoptosis assay of 2D- and 3D-cultured A549 cells. (**A**) Flow cytometry for cell apoptosis detection at 24 and 48 h after cultivation in each system. The ratio of apoptotic cells at 24 h (**B**) and 48 h (**C**) in 2D and 3D systems (*n* = 3). The results showed lower proportions of early and total apoptotic cells in the 3D system. ∗∗∗∗*P* < 0.0001; ns: *P* > 0.05.

Simultaneously, cell apoptosis between 2D and 3D systems was compared using flow cytometry. The results showed that A549 cells exhibited some degree of apoptosis after 24- and 48-h culture (Figure 5). The proportions of early and total apoptotic A549 cells in the 3D system were significantly lower than those in the 2D control (*P* < 0.0001), whereas the number of late apoptotic and necrotic cells was very low in both systems (*P* > 0.05). This suggests that A549 cells may reduce apoptosis after growth into spheroids in scaffolds. Because cells in 2D plates exhibited higher proliferation and division, excessive accumulation of cellular metabolic waste resulted in increased apoptosis. The 2D environment failed to mimic the natural structure of the tumor mass, depriving cell–cell and cell–matrix interactions and altering the cell morphology, as well as cell division [40]. Therefore, the scaffold-based design of 3D lung cancer models features a more moderate and uniform growth pattern that may better match the growth of lung cancer *in vivo*.

**Figure 6.**
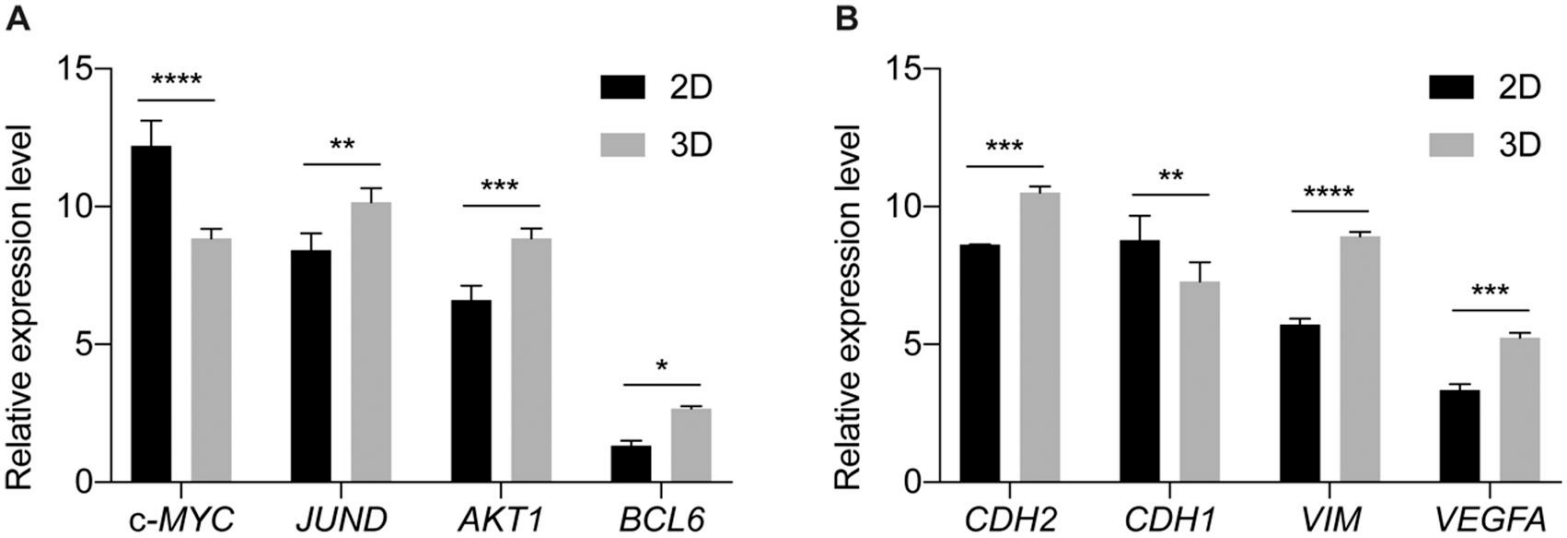
Relative expression of cell cycle regulation- and apoptosis-associated genes in 2D- and 3D-cultured A549 cells. *GAPDH* was used as the housekeeping gene. The results showed that anti-apoptotic genes were upregulated in 3D spheroids, while proliferation-promoting and pro-apoptotic gene were downregulated. A549 cells in 3D spheroids tended to undergo EMT process. ∗*P* < 0.05; ∗∗*P* < 0.01; ∗∗∗*P* < 0.001; ∗∗∗∗*P* < 0.0001.

To elucidate the results of flow cytometry, we analyzed the expression patterns of *JUND*, c*-MYC*, *BCL6* and *AKT1*, which are associated with cell cycle regulation and apoptosis in 2D and 3D systems, by qPCR (Figure 6A). The results showed that the expression of anti-apoptotic genes such as *JUND*, *BCL6* and *AKT1* was significantly higher in the scaffold-cultured A549 spheroids than in the 2D cells. In contrast, the expression of the proliferation-promoting and pro-apoptotic gene *c-MYC* was upregulated in the 2D cells compared with the spheroids in the scaffolds. More interestingly, the expression of genes associated with epithelial–mesenchymal transition (EMT), such as *CDH1 (E-cadherin)*, *CDH2 (N-cadherin)*, *VIM* and *VEGFA*, was also specifically regulated between 2D and 3D systems (Figure 6B). The epithelial cell marker *CDH1 (E-cadherin)* was found to be downregulated, whereas the mesenchymal cell markers *CDH2 (N-cadherin)*, *VIM* and *VEGFA* were upregulated in 3D spheroids compared with 2D cells. Previous studies on lung cancer models have also shown that Matrigel- or hydrogel-based scaffolds can enhance cell proliferation and induce EMT [12]. However, to the best of our knowledge, this is the first time that lung cancer cells were grown on 3D polyurethane scaffolds.

These results suggest that A549 spheroids in WBPU scaffolds exhibit higher levels of EMT, greater cell invasiveness, and higher resistance to apoptosis than 2D cells.

### 3D spheroids better match tumor xenografts

**Figure 7.**
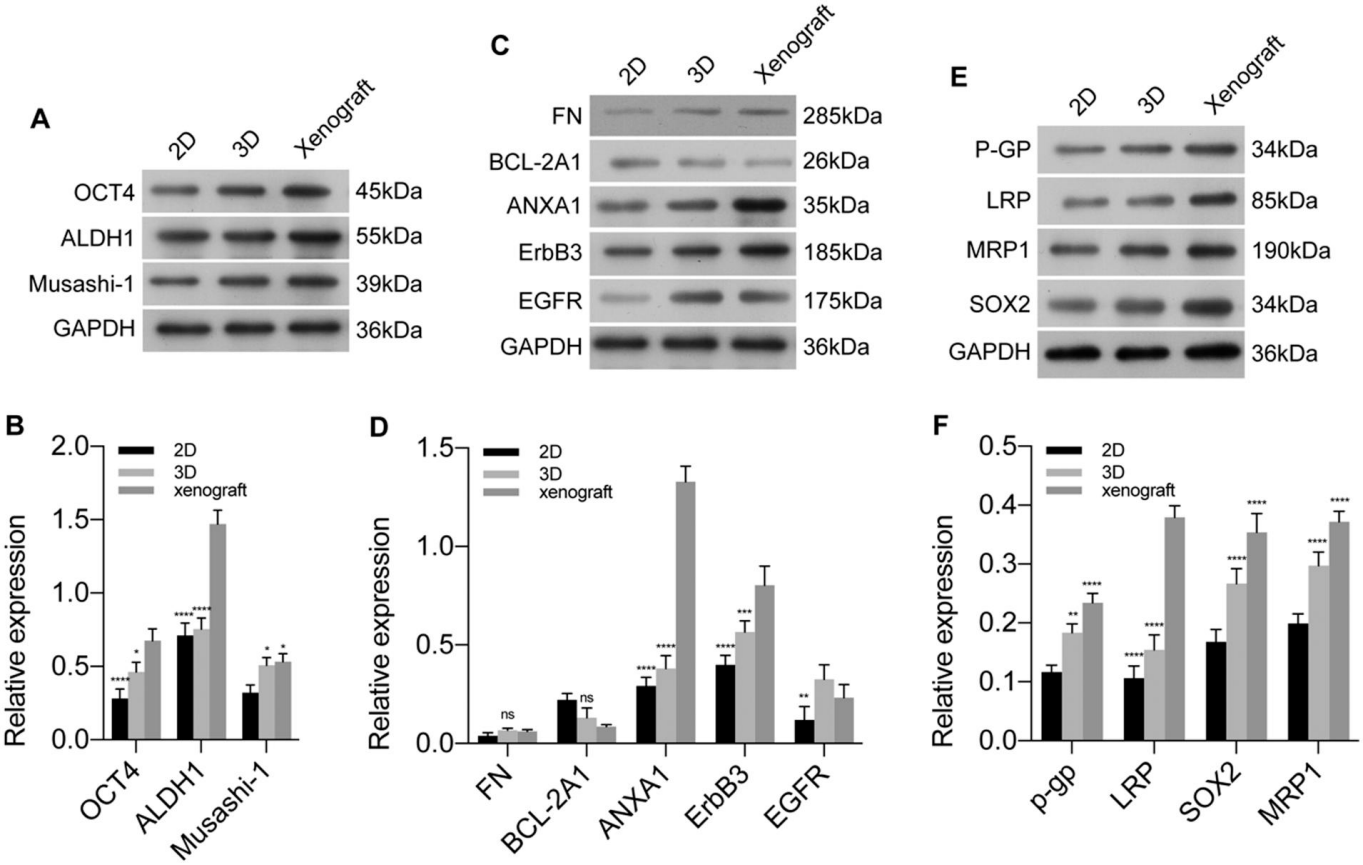
The effects of 3D microenvironment on the regulation of protein involved in stem cell characteristics (**A**, **B**), cell proliferation, invasion (**C**, **D**) and drug resistance (**E**, **F**). The results showed that A549 cells in 3D spheroids presented a similar expression pattern to the *in vivo* tumor model. ∗*P* < 0.05; ∗∗*P* < 0.01; ∗∗∗*P* < 0.001; ∗∗∗∗*P* < 0.0001; ns: *P* > 0.05.

To further evaluate 3D cultures in WBPU scaffolds, we examined relevant proteins in subcutaneous xenograft tumors in nude mice, spheroids cultured in scaffolds, and cells cultured in 2D plates by WB. These proteins are markers of tumor stem cells, cell proliferation, apoptosis, invasion and tumor resistance, which are involved in lung cancer onset and progression and can indicate the response to treatment [41, 42]. As shown in Figure 7, the expression of tumor stem cell markers OCT4, ALDH1 and Musashi-1 was significantly increased in 3D spheroids and xenograft tumors compared with cells cultured in 2D plates (Figure 7A and B), whereas the apoptosis-related protein BCL-2A1 was significantly downregulated (Figure 7C and D). The expression levels of cell proliferation and invasion-related proteins FN, ANXA1, ErbB3 and EGFR were significantly increased in the 3D spheroid and xenografts (Figure 7C and D). The expression levels of drug resistance proteins P-GP, LRP, MRP1 and SOX2 were also significantly increased in the 3D spheroids and xenografts (Figure 7E and F), indicating that the tumor cells in the 3D model had a high level of drug resistance. These results suggest that the microenvironment of the WBPU scaffold may regulate the expression levels of related proteins in the cells and that the expression levels of these proteins are similar to those of *in vivo* tumors. These results also suggest that current 2D lung cancer models have limited ability to mimic the complexity of native tumor tissue and that scaffold-based models appear to better recapitulate the characteristics of the tumor microenvironment. Therefore, scaffold-based 3D lung cancer models may be more suitable for preclinical drug screening studies.

### Modeling targeted drug treatments using scaffold-cultured 3D spheroids

The development of targeted therapies for lung cancer is essential to prevent recurrence and improve patient prognosis. In recent decades, nanocarriers have been widely used in antitumor drug delivery studies. Studies based on 2D cells have shown that nanocarriers offer excellent drug delivery and drug release advantages to tumor cells [32, 33]. However, the antitumor efficacy of these nanocarriers is significantly compromised *in vivo* models [14]. Therefore, it is of great importance to develop a suitable *in vitro* drug screening model. In the present study, the drug delivery and antitumor efficacy of nanocarrier-loaded chemotherapeutic agents developed in our previous work [28, 29] were investigated in a lung cancer model established on WBPU scaffold.

**Figure 8.**
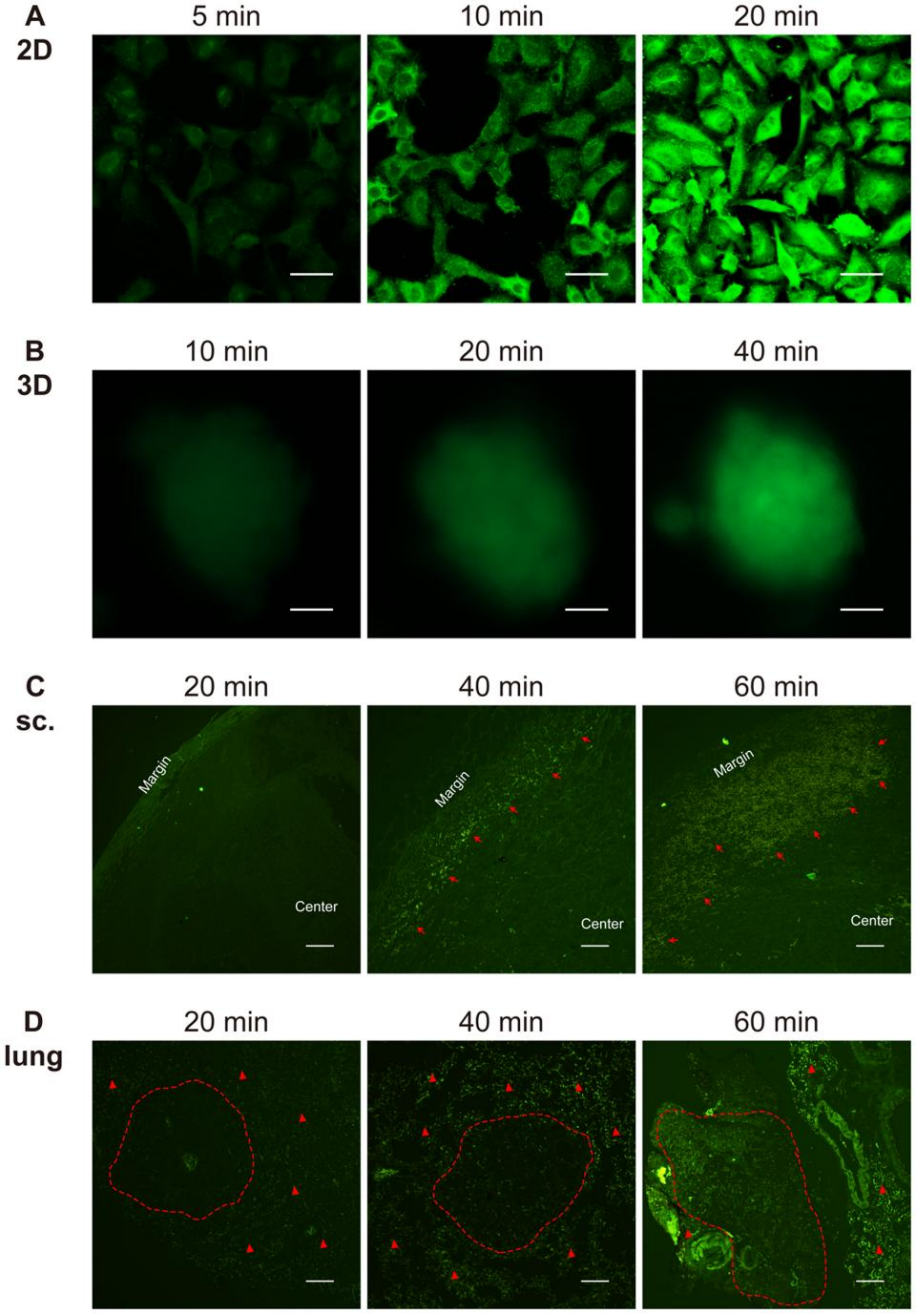
The penetration and distribution of positively charged nanocarriers in 2D-cultured cells (**A**, scale bar 20 μm), 3D spheroids (**B**, scale bar 200 μm), subcutaneous xenograft tumors (sc.) (**C**, internalization of G8mE1900-FITC micelles are indicated by red arrows, scale bar 200 μm), and *in situ* lung cancer models (**D**, nodules are circled by the red dotted line, red triangles indicate the accumulation and distribution of G8mE1900-FITC micelles in the lung interstitial, scale bar 200 μm). the results showed that positive nanocarriers penetrated and distributed faster in 2D-cultured cells than in 3D spheroids and *in vivo* models, with 3D spheroids showing a similar pattern to the *in vivo* models.

To investigate the uptake of nanocarriers in 3D lung cancer models on WBPU scaffolds, G8mE1900-FITC micelles with positively charged surfaces (Table 1) were added in different culture systems and the distribution of micelles in each model was observed under fluorescence microscope. Simultaneously, the G8mE1900-FITC micelles were injected via the tail vein, and the distribution of the micelles in subcutaneous and *in situ* lung cancer models was observed. As shown in Figure 8, G8mE1900 micelles gradually accumulated in A549 cells cultured in 2D and 3D systems and in the *in vivo* model, and the green fluorescence increased with time. The fluorescence signal accumulated more rapidly in A549 cells cultured in the 2D system and was significantly increased at 20 min after administration (Figure 8A), whereas spheroids showed slightly more visible fluorescence until 40 min after administration (Figure 8B). In the *in vivo* model, the fluorescence signal accumulated more slowly from the margin to the center of the subcutaneous xenograft and from peritumoral area to the metastatic nodules of the *in situ* lung cancer (Figure 8C and D). Furthermore, the green fluorescence was not visible at the *in vivo* tumor site until 40 min after administration. These results indicate that the penetration of the G8mE1900 nanoprobe in the *in vitro* 3D tumor model is significantly lower than that in the A549 cells grown in 2D, which is consistent with the penetration pattern of the drug in the *in vivo* model.

**Figure 9.**
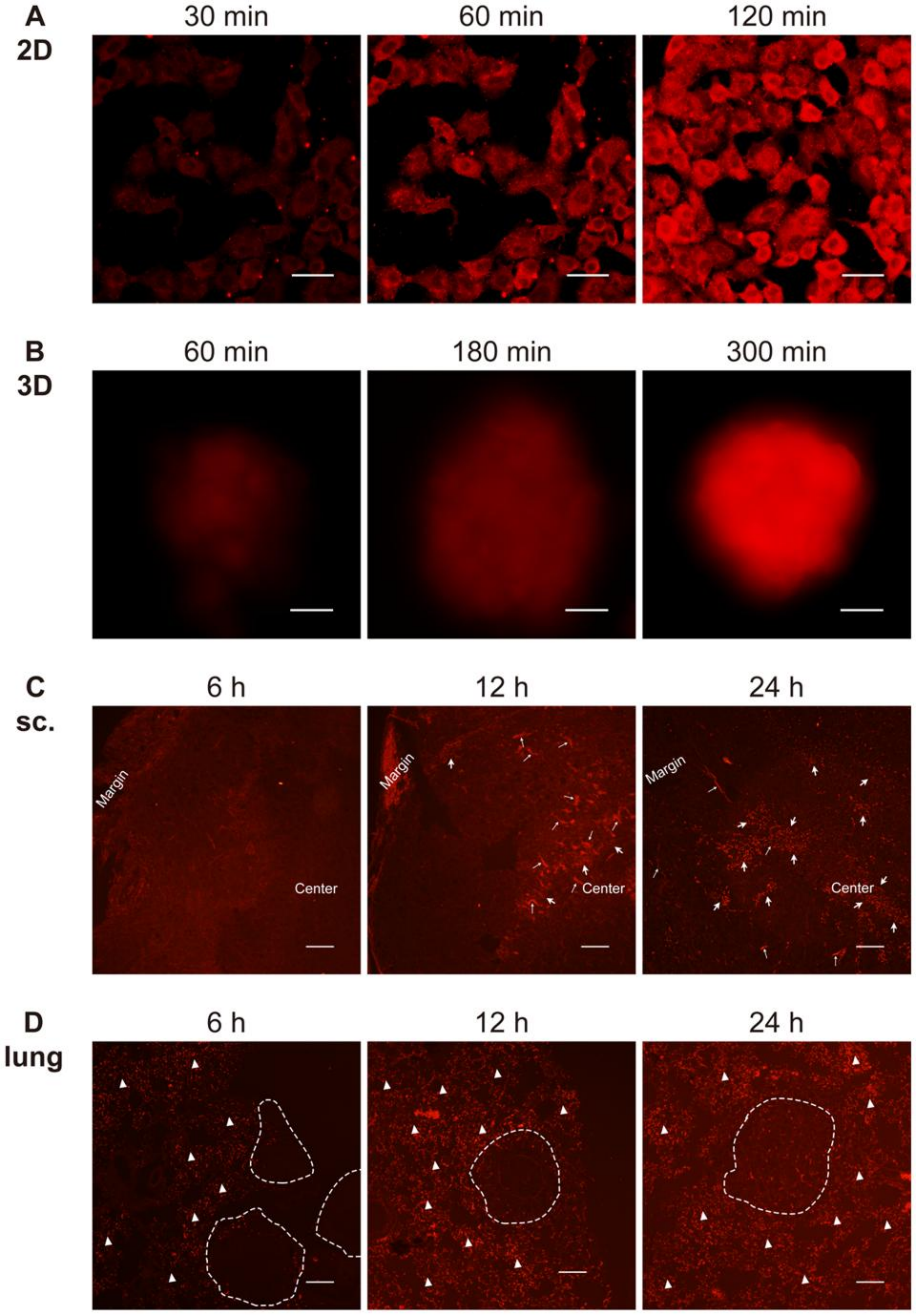
The penetration of negatively charged nanocarriers in 2D-cultured cells (**A**, scale bar 20 μm), 3D spheroids (**B**, scale bar 200 μm), subcutaneous xenograft tumors (sc.) (**C**, internalization of GFHPM-DOX micelles are indicated by thick white arrows, thin white arrows indicate the blood vessels, scale bar 200 μm), and *in situ* lung cancer models (**D**, nodules are circled by the white dotted line, white triangles indicate the accumulation and distribution of GFHPM-DOX micelles in the lung interstitial, scale bar 200 μm). The results showed that negative nanocarriers also penetrated and distributed faster in 2D-cultured cells than in 3D spheroids and *in vivo* models, with 3D spheroids showing a similar pattern to the *in vivo* models.

In addition, the distribution of surface negatively charged GFHPM delivery systems (Table 1) in different lung cancer models was also explored. As shown in Figure 9, similar to G8mE1900-FITC micelles, GFHPM-DOX micelles gradually accumulated in A549 cells cultured in 2D and 3D systems and *in vivo* models, with a progressive increase in red fluorescence. However, the accumulation of GFHPM-DOX micelles in each model was significantly slower than that of G8mE1900-FITC micelles. The fluorescence signal accumulated in A549 cells cultured in the 2D system was significantly increased 120 min after administration (Figure 9A), whereas spheroids showed slightly increased visible fluorescence until 180 min and clearly visible at 300 min after administration (Figure 9B). In the *in vivo* model, the red fluorescence signal was not visible at the tumor site until 12 h and showed a steady increase from 12 to 24 h after administration (Figure 9C and D). The distribution pattern of the GFHPM-DOX in both *in vivo* lung cancer models was similar to that of the G8mE1900-FITC micelles. In summary, tumor cell internalization of GFHPM-DOX micelles is slower than that of G8mE1900-FITC, and penetration of GFHPM-DOX micelles in the 3D tumor model is significantly lower than that in 2D-grown A549 cells, mimicking the drug penetration pattern in the *in vivo* models.

**Figure 10.**
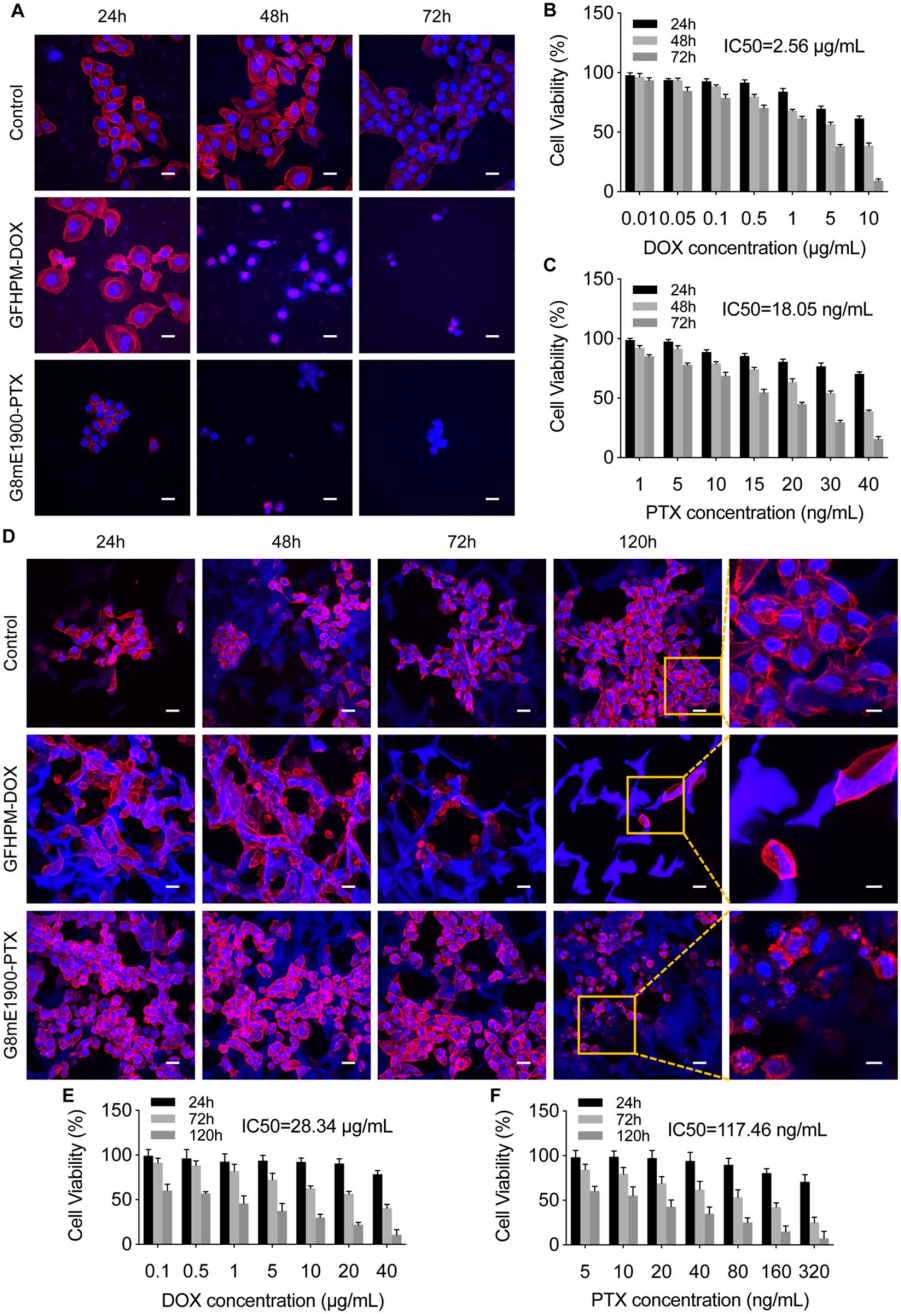
Pharmacodynamic evaluation of drug delivery nanocarriers on scaffold-derived 3D lung cancer models. (**A**) Morphology of 2D cells treated with GFHPM-DOX and G8mE1900-PTX micelles at 24, 48 and 72 h, respectively. Scale bar 20 μm. (**B**) Cell viability of 2D cells treated with GFHPM-DOX micelles (*n* = 3). (**C**) Cell viability of 2D cells treated with G8mE1900-PTX micelles (*n* = 3). (**D**) Morphology of 3D spheroids treated with GFHPM-DOX and G8mE1900-PTX micelles at 24, 48, 72 and 120 h, respectively. Scale bars 20 μm for low-magnification field and 10 μm for high-magnification field. (**E**) Cell viability of 3D spheroids treated with GFHPM-DOX micelles (*n* = 3). (**F**) Cell viability of 3D spheroids treated with G8mE1900-PTX micelles (*n* = 3). The results showed that the 3D lung cancer model is more tolerant to antitumor drugs than the conventional 2D model cells.

In view of this, we hypothesize that 3D lung cancer models constructed with polyurethane scaffolds may be more suitable for nano-drug screening. Subsequently, the antitumor effects of the two drug-loaded micelles were tested in different lung cancer models using positively charged G8mE1900 micelles loaded with paclitaxel and negatively charged GFHPM micelles loaded with doxorubicin. The results showed that the activity of A549 cells in 2D culture was significantly inhibited over time after the addition of the drug-loaded micelles compared with the control group (Figure 10A–C). The morphology of the cells changed, with most cells becoming roundish, detached and floated (Figure 10A). The IC50 of GFHPM-DOX for A549 cells cultured in 2D was approximately 2.56 μg/ml (Figure 10B), and the IC50 of G8mE1900-PTX for *in vitro* 2D culture was approximately 18.05 ng/ml (Figure 10C). Simultaneously, serial concentrations of drug-loaded micelles were added to the *in vitro* 3D lung cancer model cultures. As illustrated in Figure 10D–F, cellular activity gradually declined over time and cell morphology changed, with most cells becoming rounded and detached from the scaffold (Figure 10D). The IC50 of GFHPM-DOX for the *in vitro* 3D tumor model was approximately 28.34 μg/ml, which was 11.07 times higher than that for the 2D cultured cells (Figure 10E). The IC50 of G8mE1900-PTX for the *in vitro* 3D tumor model was approximately 117.46 ng/ml, which was 6.51 times higher than that for the 2D cultured cells (Figure. 10F). These results indicate that the *in vitro* 3D lung cancer model is more tolerant to antitumor drugs than the conventional 2D model cells. As a reference, the subcutaneous and *in situ* lung cancer models were treated with drug-loaded micelles at a dose based on the 3D model to observe the antitumor effect and safety of these drugs. As shown in Supplementary Figures S2–S5, tumor volume and weight of subcutaneous xenograft tumors in the GFHPM-DOX and G8mE1900-PTX treatment groups were significantly lower than those in the control group (Supplementary Figures S2A–C, S4A–C), and significant necrosis and apoptosis were observed within the tumor tissues in the treatment groups (Supplementary Figures S2D and S4D). Similarly, the number of *in situ* lung cancer nodules was significantly reduced in both treatment groups compared to the control group (Supplementary Figures S3A and B, S5A and B), and necrosis and apoptosis were more pronounced within the tumor tissue (Supplementary Figures S3C and S5C). These results showed that the 3D lung cancer model was more tolerant to the nanoparticulate drug delivery systems than the conventional 2D-cultured cells and better matched the tolerance dose in the *in vivo* models.

The above results indicate that the 3D lung cancer model based on WBPU scaffolds could be used as a screening system for *in vivo* drug delivery.

**Table 1.** Characteristics of G8mE1900 and GFHPM micelles

	Size (d nm)	PDI	Zeta potential (mV)	LC (%)	EE (%)
G8mE1900	61.77 ± 1.24	0.233	29.83 ± 1.03	18	99
GFHPM	78.2 ± 0.7	0.333	−16.13 ± 1.06	14.2	71

EE, drug encapsulation efficiency; LC, drug loading content.

## Conclusions

Lung cancer is a challenging disease that threatens public health, and there is an urgent need to develop effective therapies. We have successfully constructed an *in vitro* 3D lung cancer model based on the WBPU scaffold. We have shown that this model could better match the *in vivo* model in terms of tumor microenvironment, drug permeability, and drug sensitivity, and thus may be used as a model for screening antitumor drugs for lung cancer. Before moving to animal testing, the 3D scaffold-based lung cancer model could further narrow down the scope, dosage form, and dose of screening drugs to be used. Due to the obvious heterogeneity and different response of lung cancer cells to drugs, appropriate substrates need to be developed. Therefore, the construction of preclinical tumor models should develop appropriate substrates that could be used as individualized screening systems to find the most promising treatment options for clinical patients.

## Supplementary Material

rbad091_Supplementary_Data
