# Supplementary material for: Polyurethane scaffold-based 3D lung cancer model recapitulates in vivo tumor biological behavior for nanoparticulate drug screening
Source: Regen Biomater. 2023 Oct 25;10:rbad091. doi: 10.1093/rb/rbad091 (PMC10641150; doi:10.1093/rb/rbad091)
Supplement: rbad091_Supplementary_Data [file rbad091_supplementary_data.docx]

**Supporting information**

Polyurethane scaffold-based 3D lung cancer model recapitulates *in vivo* tumor biological behavior for nanoparticulate drug screening

Lu Sun^1^, Xiaofei Wang^2^, Yushui He^2^, Boran Chen^3^, Baoyin Shan^3^, Jinlong Yang^3^, Ruoran Wang^3^, Xihang Zeng^3^, Jiehua Li^2^, Hong Tan^2,^ *, Ruichao Liang^3,^ *

^1^Department of Targeting Therapy & Immunology; Department of Radiation Oncology, Cancer Center, West China Hospital, Sichuan University, Chengdu 610041, People's Republic of China

^2^Department of Medical Polymer Materials, College of Polymer Science and Engineering, State Key Laboratory of Polymer Materials Engineering, Sichuan University, Chengdu 610065, People's Republic of China

^3^Department of Neurosurgery, West China Hospital, Sichuan University, Chengdu 610041, People's Republic of China

*Corresponding author.

E-mail: dr_liangruichao@scu.edu.cn (Dr. Ruichao Liang); [hongtan@scu.edu.cn](mailto:hongtan@scu.edu.cn) (Prof. Hong Tan)


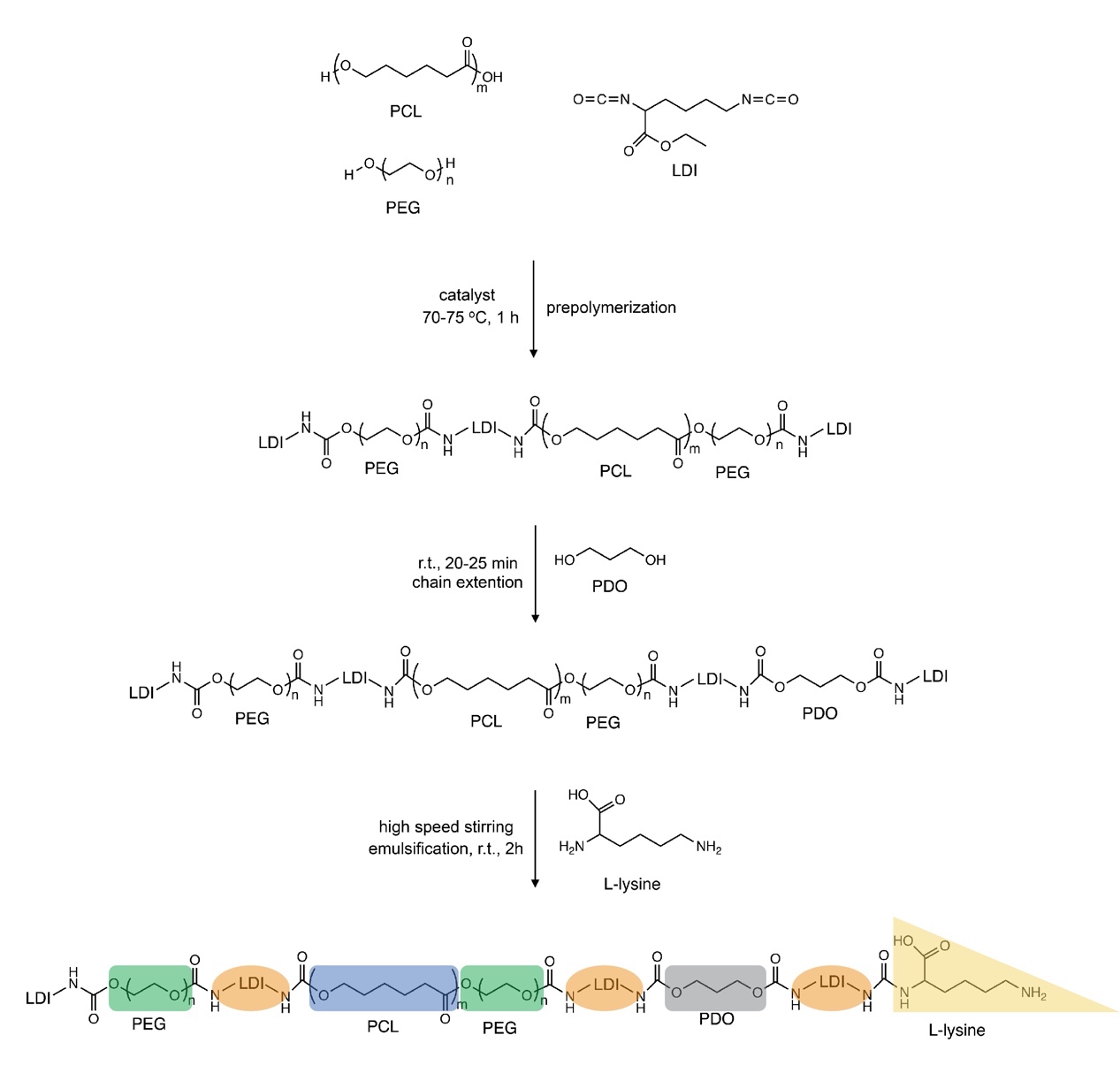


Fig. S1 Schematic of the preparation of WBPU emulsions.


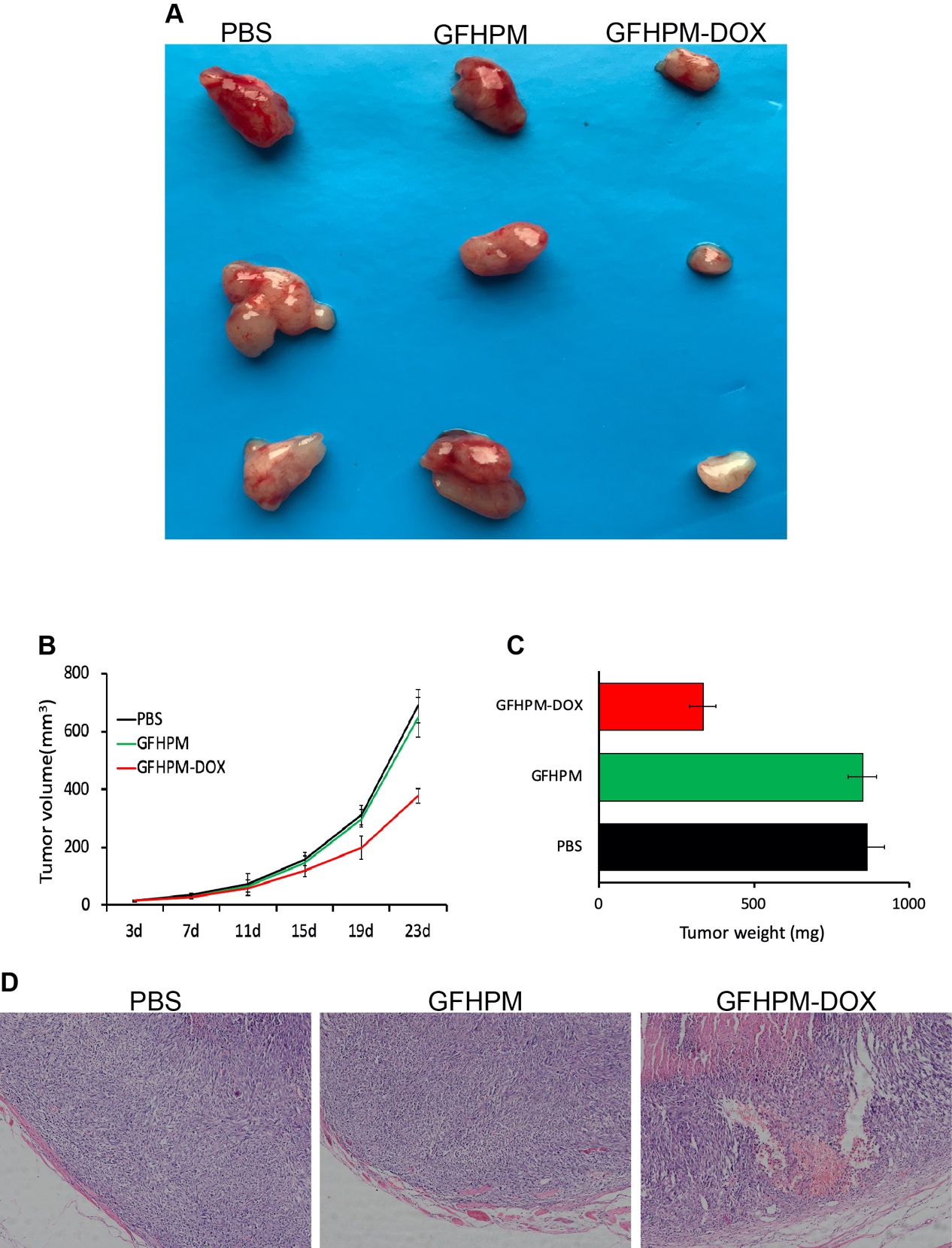


Fig. S2 Antitumor effects of GFHPM-DOX micelles in subcutaneous xenograft model. (A) Tumor morphology at endpoint of treatment (n=3). (B) Tumor volume profile of each treating group (n=3). (C) Tumor weight at endpoint of treatment (n=3). (D) H&E image of harvested tumor.


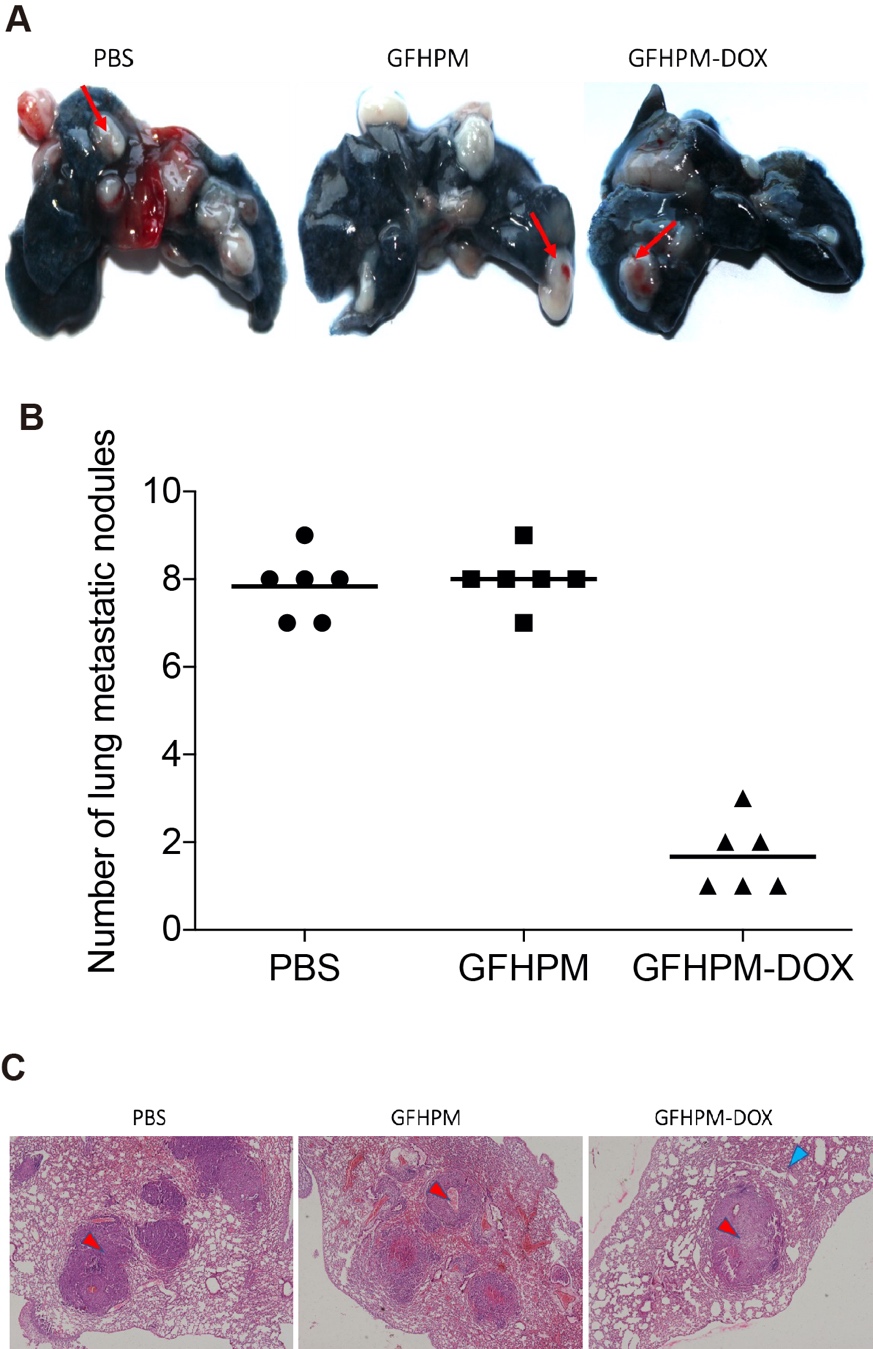


Fig. S3 Antitumor effects of GFHPM-DOX micelles in in situ lung cancer model. (A) Lung tumor nodules morphology at endpoint of treatment (n=3). (B) Number of lung cancer nodules in each treating group (n=3). (C) H&E image of harvested lung with tumor, red arrows showed solid portion of the tumor.


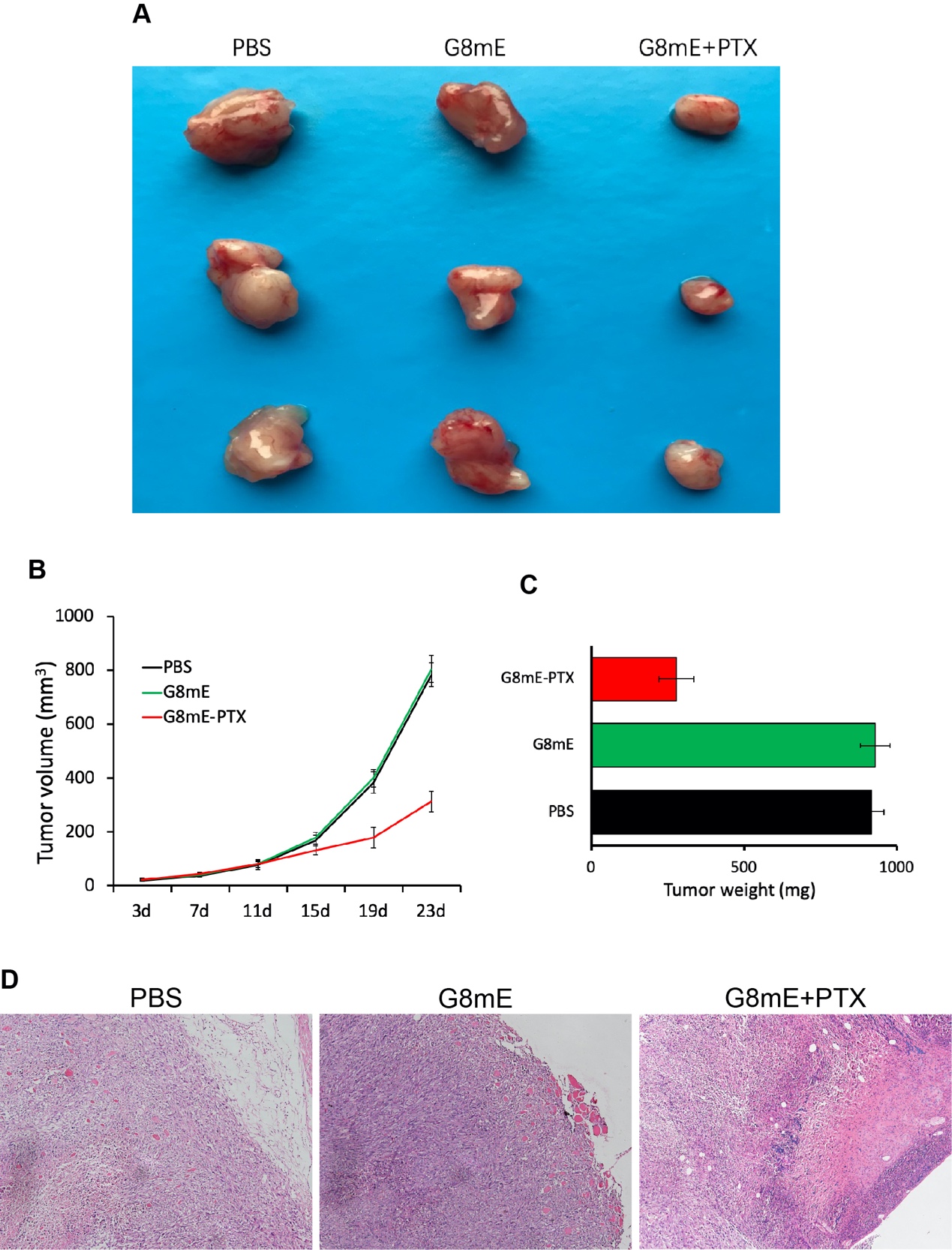


Fig. S4 Antitumor effects of G8mE-PTX micelles in subcutaneous xenograft model. (A) Tumor morphology at endpoint of treatment (n=3). (B) Tumor volume profile of each treating group (n=3). (C) Tumor weight at endpoint of treatment (n=3). (D) H&E image of harvested tumor.


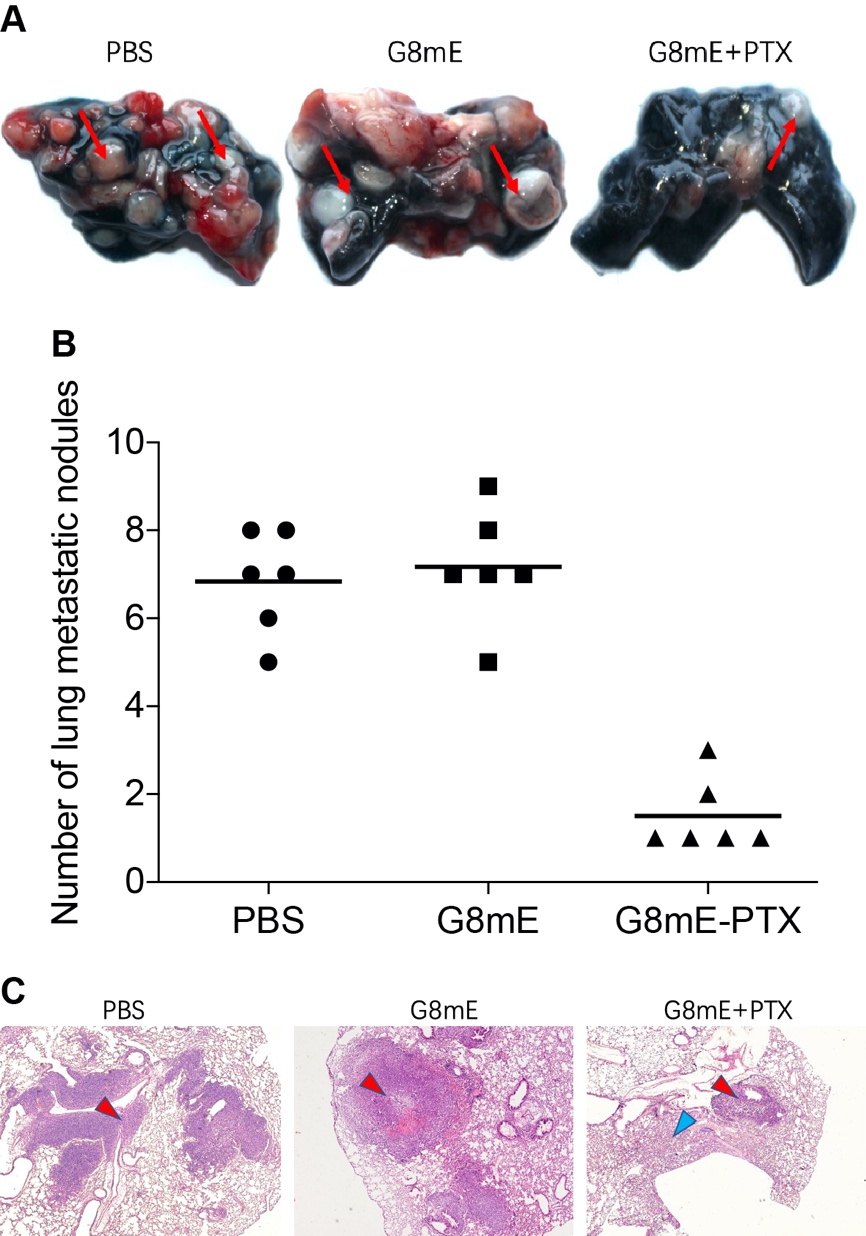


Fig. S5 Antitumor effects of G8mE-PTX micelles in in situ lung cancer model. (A) Lung tumor nodules morphology at endpoint of treatment (n=3). (B) Number of lung cancer nodules in each treating group (n=3). (C) H&E image of harvested lung with tumor, red arrows showed solid portion of the tumor.

**Table S1** Primer sequences (5' -> 3') for qPCR reactions

| Gene | Forward | Reverse |
| --- | --- | --- |
| *GAPDH* | GGAGCGAGATCCCTCCAAAAT | GGCTGTTGTCATACTTCTCATGG |
| *JUND* | TCATCATCCAGTCCAACGGG | TTCTGCTTGTGTAAATCCTCCAG |
| *c-MYC* | GGCTCCTGGCAAAAGGTCA | CTGCGTAGTTGTGCTGATGT |
| *BCL6* | GGAGTCGAGACATCTTGACTGA | ATGAGGACCGTTTTATGGGCT |
| *AKT1* | AGCGACGTGGCTATTGTGAAG | GCCATCATTCTTGAGGAGGAAGT |
| *CDH1* | CGAGAGCTACACGTTCACGG | GGGTGTCGAGGGAAAAATAGG |
| *CDH2* | TCAGGCGTCTGTAGAGGCTT | ATGCACATCCTTCGATAAGACTG |
| *VIM* | GACGCCATCAACACCGAGTT | CTTTGTCGTTGGTTAGCTGGT |
| *VEGFA* | AGGGCAGAATCATCACGAAGT | AGGGTCTCGATTGGATGGCA |
